# Integrating Family Involvement in Neuro-Intensive Care: A Pathway to Enhanced Family Satisfaction in Traumatic Brain Injury (TBI) Management

**DOI:** 10.7759/cureus.74748

**Published:** 2024-11-29

**Authors:** Sindu K Mathew, S Aruna, Ramesh C Vasudevan, Vivek Visweswaran, Porkodi Arjunan, Binuja Panachingal, Mibi Jyothi

**Affiliations:** 1 Medical-Surgical Nursing, Sri Ramachandra Institute of Higher Education and Research, Chennai, IND; 2 Community Health Nursing, Sri Ramachandra Institute of Higher Education and Research, Chennai, IND; 3 Neurological Surgery, Tellicherry Co-operative Hospital, Thalassery, IND; 4 Neurological Surgery, Sri Ramachandra Institute of Higher Education and Research, Chennai, IND; 5 Nursing, College of Applied Medical Sciences, King Faisal University, Al-Ahsa, SAU; 6 Psychiatric Nursing, College of Nursing Thalassery, Thalassery, IND; 7 Medical-Surgical Nursing, College of Nursing Thalassery, Thalassery, IND

**Keywords:** communication, family icu syndrome, family involvement in care, family satisfaction, family satisfaction in the intensive care unit 24r (fs-icu 24r), holistic neurocritical care model, neuro-intensive care, shared decision-making in the icu, staff burnout, traumatic brain injury critical care

## Abstract

Introduction

Traumatic brain injury (TBI) is a major health concern due to its poor clinical and functional outcome and its impact on family homeostasis. Often, the stressed and anxious family is not involved in the care process until the rehabilitation phase. This study aimed to evaluate the integration of family involvement in the neuro-intensive care of TBI patients on family satisfaction.

Materials and methods

This pre-test-post-test quasi-experimental study involved 70 families of moderate and severe TBI patients, divided equally into experimental and control groups. A guideline, Family Involvement in the Care of TBI (FIC-TBI), was developed. The key elements of this guideline were structured communication, participation in basic care, and shared decision-making. FIC-TBI was integrated into the critical care management of TBI. The impact of FIC-TBI was evaluated using the Family Satisfaction in the ICU Care Unit 24R (FS-ICU 24R) tool.

Results

Data analysis was done through paired and unpaired t-tests as well as analysis of covariance (ANCOVA). The results showed statistically significant differences in the post-test family satisfaction scores between the experimental and control groups. The adjusted mean satisfaction scores of components such as satisfaction with care (F(1, N-2)=984.63; p<0.001), satisfaction with decision-making (F(1, N-2)=1489.63; p<0.001), and overall satisfaction (F(1, N-2)=712.87; p<0.001) were higher in the experimental group. The study found a significant correlation between family satisfaction levels and variables such as gender and education.

Conclusions

This study was done with the objective to evaluate the impact of integration of family involvement into the neuro-intensive care of TBI on family satisfaction. The findings demonstrated that the FIC-TBI guideline improved satisfaction across multiple dimensions, such as care, communication, decision-making, and overall experience. The study also found a significant correlation between family satisfaction and gender and education. This study emphasized the importance of early family involvement in critical care settings as a vital component of holistic TBI management.

## Introduction

Traumatic brain injury (TBI) is a significant and persistent challenge within healthcare systems worldwide. This heightened concern stems from several critical factors, including its high mortality and morbidity rates, the disproportionate impact on young adults, poor clinical and functional recovery outcomes, and the profound disruption it causes to family life and quality of living [1]. The complexity of TBI management necessitates specialized care, typically provided within neuro-intensive care units (neuro-ICUs). Studies highlight that TBI is one of the most frequently encountered conditions in neuro-ICUs, often accompanied by a surge of family members seeking information and reassurance about their loved ones [2,3].

The emergency nature of TBI, coupled with the urgent and complex medical decisions required, often leaves families unprepared to navigate such a critical health crisis. Factors such as limited visitation opportunities, fear of losing their loved one, and the overwhelming stress of the situation significantly contribute to their emotional distress [4,5]. These challenges frequently result in heightened interactions between families and neuro-ICU staff. However, communication in these scenarios is often brief, focused primarily on clinical facts, and leaves little opportunity for families to express their emotions or seek psychological support from the staff [6-10].

While TBI treatment has traditionally prioritized clinical recovery, growing evidence emphasizes the importance of addressing the psychological and emotional well-being of both patients and their families as integral to successful outcomes [11,12]. Families increasingly desire greater involvement in the care process, and their interactions with healthcare providers play a pivotal role in shaping treatment experiences and outcomes [13-15]. Research indicates that when families are actively involved in essential care within the neuro-ICU, both patient recovery and family satisfaction improve significantly [16,17].

The introduction of protocols that emphasize effective communication, structured family involvement, and shared decision-making is essential for creating a supportive care environment. Such protocols not only uphold the rights of patients but also alleviate the psychological burden on families, fostering trust and satisfaction in the care process [18,19]. Family involvement extends beyond providing emotional support. It includes participation in basic patient care, engaging in care planning discussions, and assisting the healthcare team in tailoring interventions based on the patient's preferences. This collaboration not only empowers families to cope with stress but also helps multidisciplinary teams deliver more patient-centered care [20,21]. Studies have shown that involving families in communication and care planning significantly reduces their levels of anxiety and depression while enhancing their overall experience in the ICU [22,23]. Additionally, family involvement ensures continuity of care, which is particularly vital for TBI patients, many of whom face prolonged physical and cognitive rehabilitation challenges [24,25].

In recent years, family satisfaction has emerged as a critical measure of quality in critical care settings. Research consistently demonstrates that improved communication, shared decision-making, and active family participation are associated with higher levels of satisfaction. A meta-analysis of family-centered care models revealed notable reductions in stress levels and enhanced satisfaction among families involved in the care process [26-28].

This study explores the impact of structured family involvement in the care of TBI patients on family satisfaction. It forms part of a larger initiative aimed at developing and evaluating the Neuro Intensive Care Protocol for TBI (NICP-TBI). A key component of this protocol is the Family Involvement in the Care of TBI (FIC-TBI), which provides clear guidelines for structured communication, participation in basic care, and shared decision-making [29,30]. By integrating family involvement into TBI management, this study seeks to advance understanding and improve outcomes in neurocritical care settings.

## Materials and methods

This prospective pre-test-post-test quasi-experimental study was done in the neuro-ICUs of Tellicherry Co-operative Hospital and Indira Gandhi Co-operative Hospital Thalassery between June 2022 and May 2024. The purpose of the study was to evaluate the impact of the integration of family involvement in the care of TBI patients on family satisfaction. The study involved 70 families of TBI patients, equally distributed to experimental and control groups. This study was part of a broader study titled "Development, validation, and evaluation of a neuro intensive care protocol for TBI (NICP -TBI)". FIC-TBI was one of the components of the NICP-TBI. The study protocol was approved by the Institutional Ethics Committee (IEC) of Sri Ramachandra Institute of Higher Education and Research (approval number: IEC/21/OCT/165/69). All samples were given participant information sheets and informed consent.

Selection of sample

Family members of adult patients with moderate (Glasgow Coma Scale (GCS) score 9-12) or severe (GCS score ≤8) TBI undergoing treatment in the neuro-ICU for more than 48 hours were considered eligible samples. Convenience sampling was adopted as the sampling technique.

Description of intervention

FIC-TBI Guideline

The broader components of the guideline were structured communication, participation in basic care, and shared decision-making. The FIC-TBI model framework for neuro-ICU TBI management is presented in Table 1.

**Table 1:** FIC-TBI model framework for neuro-ICU TBI management FIC-TBI: Family Involvement in the Care of TBI; neuro-ICU: neuro-intensive care unit; TBI: traumatic brain injury

SI. no.	Pillars	Components	Activities
1	Pillar 1	Family orientation and education	Family orientation. Assessment of readiness and capability. Education and training. Roles and responsibilities
2	Pillar 2	Active participation and support	Involvement in daily care. Communication and decision-making. Emotional and psychological support
3	Pillar 3	Feedback and evaluation	Feedback. Document involvement. Team meeting

Data collection and study process

In the study, 35 participants in the control group received standard information concerning their relatives admitted to the neuro-ICU. Family satisfaction was initially assessed 24 hours post-admission using the Family Satisfaction in the ICU Care Unit 24R (FS-ICU 24R) scale. Routine care practices and family communication processes were maintained throughout the neuro-ICU stay, with a second assessment conducted upon patient transfer from the neuro-ICU.

For the experimental group, a structured guideline titled "Family Involvement in the Care of TBI (FIC-TBI)" was introduced to the neuro-ICU's multidisciplinary healthcare team before study initiation. This intervention was launched through a workshop, where neuro-ICU professionals practiced the care model with standardized patient and family scenarios and were evaluated using a scoring sheet. An initial satisfaction assessment was performed 24 hours after neuro-ICU admission. Subsequently, the FIC-TBI guideline was systematically integrated into TBI management. A final satisfaction assessment using FS-ICU 24R was conducted after each patient's neuro-ICU stay.

## Results

Description of sample characteristics in the experimental and control groups

The study included a total of 70 participants, equally distributed between the experimental group (n=35) and the control group (n=35). A comparative analysis of the baseline characteristics revealed that both groups were generally comparable across most demographic variables, such as gender, age distribution, relationship with the patient, and previous ICU experience. However, a statistically significant difference was observed in the educational status of participants between the two groups (p=0.03). This indicates that the two groups were otherwise homogenous, with education being the only exception. The detailed breakdown of the characteristics is presented in Table 2.

**Table 2:** Comparison of baseline characteristics between the experimental and control groups ICU: intensive care unit *: significant (p<0.05)

Sample characteristics	Experimental group (n=35), f (%)	Control group (n=35), f (%)	χ^2^	df	P-value
Gender					
Male	21 (60)	17 (48.6)	0.92	1	0.33
Female	14 (40)	18 (51.4)			
Age					
20-29 years	7 (20)	11 (31.4)	3.75	4	0.44
30-39 years	8 (22.9)	11 (31.4)			
40-49 years	9 (25.7)	8 (22.9)			
50-59 years	8 (22.9)	4 (11.4)			
60-69 years	3 (8.6)	1 (2.9)			
Relationship with the patient					
Husband/wife	7 (20)	8 (22.9)	0.48	4	0.97
Father/mother	3 (8.6)	3 (8.6)			
Sister/brother	12 (34.3)	13 (37.1)			
Daughter/son	11 (31.4)	10 (28.6)			
Relatives	2 (5.7)	1 (2.9)			
Have you previously stayed with a family member in the ICU?					
Yes	31 (88.6)	30 (85.7)	0.12	1	0.72
No	4 (11.4)	5 (14.3)			
Education					
High school dropout	8 (22.9)	2 (5.7)	10.16	4	0.03^*^
High school	8 (22.9)	6 (17.1)			
Higher secondary school	11 (31.4)	20 (57.1)			
Graduation	3 (8.6)	6 (17.1)			
Post-graduation	5 (14.3)	1 (2.9)			

Comparative analysis of family satisfaction levels between the experimental and control groups

The pre-test analysis given in Table 3 indicated significant differences in family satisfaction levels between the experimental and control groups. The control group reported higher satisfaction in all domains: satisfaction with care (t=8.98; p<0.00), satisfaction with decision-making (t=5.75; p<0.00), and overall satisfaction (t=7.85; p<0.00). These findings indicate heterogeneity between the experimental and control groups.

**Table 3:** Comparison of pre-test scores of family satisfaction levels between the experimental and control groups *: significant (p<0.05)

Domain	Experimental (n=35), mean±SD		Control (n=35), mean±SD		f	t-value	P-value
Satisfaction with care	48.7±6.65		62.2±5.85		68	8.98	0.00^* ^
Satisfaction with decision-making	46.07±6.7		56.1±7.89			5.75	0.00^*^
Overall satisfaction	47.72±5.64		60.75±8.01			7.85	0.00*

Impact of integrating FIC-TBI on family satisfaction in the neuro-ICU setting

Comparative Analysis of Family Satisfaction Levels Between the Experimental and Control Groups

The results of the analysis of covariance (ANCOVA) analysis presented in Table 4 indicate statistically significant differences in adjusted post-test scores for family satisfaction between the experimental and control groups. The experimental group demonstrated significantly higher adjusted mean scores across all domains: satisfaction with care (F=984.63; p<0.00), satisfaction with decision-making (F=1489.63; p<0.00), and overall satisfaction (F=712.87; p<0.00). These findings suggest that the intervention had a statistically significant effect on enhancing family satisfaction. The large F-values confirmed the substantial effect size and indicated that the intervention accounted for a significant proportion of the variance in satisfaction levels between groups.

**Table 4:** Adjusted post-test score comparison (ANCOVA) for family satisfaction levels between the experimental and control groups ANCOVA: analysis of covariance; a: adjusted mean *: significant (p<0.05)

Variable	Experimental	Control	df	ANCOVA	P-value
	Group mean	Group mean		F	
Satisfaction with care	100.73^a^	60.74^a^	1	984.63	0.00^*^
						Satisfaction with decision-making	100.26^a^	53.95^a^	1	1489.63	0.00^*^
Overall satisfaction	103.02^a^	57.30^a^	1	712.87	0.00^*^						

Correlation Analysis Between Satisfaction Levels and Personal Demographics

The analysis in Table 5 examines the correlation between family satisfaction levels and demographic variables. Significant associations were found for gender (χ²=3.68; p=0.05) and education level (χ²=10.53; p=0.03). Female gender and those with higher education levels reported greater satisfaction. No significant correlations were observed for age (χ²=7.66; p=0.1), relationship with the patient (χ²=1.34; p=0.85), or prior ICU experience (χ²=1.14; p=0.28).

**Table 5:** Correlation analysis between family satisfaction and demographics ICU: intensive care unit *: significant (p<0.05)

Personal variables	Category	Family satisfaction level		χ^2^	df	P-value
		< median	> median			
Gender						
Male		23	15	3.68	1	0.05^*^
Female		12	20			
Age						
20-29 years		5	13	7.66	4	0.1
30-39 years		9	10			
40-49 years		9	8			
50-59 years		9	3			
60-69 years		3	1			
Relationship with the patient						
Partner		6	9	1.34	4	0.85
Father/mother		3	3			
Sister/brother		14	11			
Daughter/son		10	11			
Other		2	1			
Have you previously stayed with a family member in the ICU?						
Yes		32	29	1.14	1	0.28
No		3	6			
Education						
High school dropout		7	3	10.53	4	0.03^*^
Completed high school		11	3			
Higher secondary school		12	19			
Graduation		2	7			
Post-graduation		3	3			

## Discussion

The findings of this study indicated that structured family involvement based on the FIC-TBI guidelines significantly improved family satisfaction in care, decision-making, and overall ICU experience. The results of the study are aligned with research studies on family-centered care models in critical care, which highlighted family inclusion's benefits for both patients and their families.

This study showed a significant improvement in the care satisfaction scores in the experimental group (mean increase from 48.79 to 98.27; p<0.001). This finding is consistent with a study by Davidson et al., which emphasized how family-centered approaches addressed the psychological needs of families, reducing their anxiety and promoting a more inclusive environment [15]. Similarly, Coombs et al. found that effective communication fosters a sense of partnership between family members and healthcare providers, thereby increasing satisfaction [13].

In terms of decision-making satisfaction, our study findings align closely with the findings of a study by Curtis and White, which documented that involving family members in decision-making processes leads to greater clarity and satisfaction with care outcomes [27]. Olding et al. also highlighted the importance of family engagement in critical decisions, which is associated with improved family understanding and comfort in a critical care setting [14].

The correlation analysis in this study indicated that family satisfaction is significantly associated with demographic factors such as gender and education. This analysis indicates the importance of considering demographic factors in understanding and improving satisfaction with critical care. Mitchell and Aitken's research similarly found that demographic characteristics influence family needs and their perception of ICU care quality, and the study suggested the necessity of a structured approach to meet diverse family expectations [23]. Adams et al. echoed these findings, noting that understanding family characteristics can enhance satisfaction by facilitating customized support [24].

Study limitations

This study has certain limitations that could affect the generalizability of the results. The relatively small sample size (n=70) may limit the statistical power when applied to broader populations or different neuro-ICU settings. The use of self-reported measures through the FS-ICU 24R tool could introduce bias, as participants may feel inclined to respond positively due to perceived social expectations. To validate the statistically significant results of FIC-TBI guidelines, multicenter studies across diverse settings are needed.

## Conclusions

In this study, we investigated the impact of the integration of structured family involvement in the care of TBI patients in the neuro-ICU and its correlation with family satisfaction. A total of 70 families of TBI patients were surveyed to evaluate the effects of this approach. This study demonstrated that integrating structured family involvement using the FIC-TBI guidelines significantly enhances family satisfaction in neuro-ICU settings. This study's findings indicated that structured communication, participation in care, and shared decision-making are the key elements in improving family satisfaction across various dimensions, including care quality and decision-making processes. The correlation with demographic factors like gender and education, found in the study, highlights the need for incorporating a holistic approach and personalized family support strategies in neurocritical care. Future research should expand on the findings with larger, multicenter studies to further validate the impact on long-term patient outcomes and adapt the guidelines to diverse cultural settings.

## References

[1] Maas AI, Menon DK, Manley GT (2022). Traumatic brain injury: progress and challenges in prevention, clinical care, and research. Lancet Neurol.

[2] Lindlöf J, Turunen H, Coco K, Huhtakangas J, Verhaeghe S, Välimäki T (2024). Empowering support for family members of patients with traumatic brain injury during the acute care: insights from family members and nurses. J Adv Nurs.

[3] Linnarsson JR, Bubini J, Perseius KI (2010). A meta-synthesis of qualitative research into needs and experiences of significant others to critically ill or injured patients. J Clin Nurs.

[4] Frivold G, Slettebø Å, Dale B (2016). Family members' lived experiences of everyday life after intensive care treatment of a loved one: a phenomenological hermeneutical study. J Clin Nurs.

[5] Baumhover NC, May KM (2013). A vulnerable population: families of patients in adult critical care. AACN Adv Crit Care.

[6] Abdul Halain A, Tang LY, Chong MC, Ibrahim NA, Abdullah KL (2022). Psychological distress among the family members of intensive care unit (ICU) patients: a scoping review. J Clin Nurs.

[7] Whiffin CJ, Gracey F, Ellis-Hill C (2021). The experience of families following traumatic brain injury in adult populations: a meta-synthesis of narrative structures. Int J Nurs Stud.

[8] Davidson JE, Jones C, Bienvenu OJ (2012). Family response to critical illness: postintensive care syndrome-family. Crit Care Med.

[9] Seaman JB, Arnold RM, Scheunemann LP, White DB (2017). An integrated framework for effective and efficient communication with families in the adult intensive care unit. Ann Am Thorac Soc.

[10] Salins N (2015). Measuring family satisfaction in an Indian intensive care unit. Indian J Crit Care Med.

[11] Seyedfatemi N, Mohammadi N, Hashemi S (2020). Promoting patients health in intensive care units by family members and nurses: a literature review. J Educ Health Promot.

[12] Anderson MI, Simpson GK, Daher M, Matheson L (2015). The relationship between coping and psychological adjustment in family caregivers of individuals with traumatic brain injury: a systematic review. Annu Rev Nurs Res.

[13] Coombs M, Puntillo KA, Franck LS, Scruth EA, Harvey MA, Swoboda SM, Davidson JE (2017). Implementing the SCCM family-centered care guidelines in critical care nursing practice. AACN Adv Crit Care.

[14] Olding M, McMillan SE, Reeves S, Schmitt MH, Puntillo K, Kitto S (2016). Patient and family involvement in adult critical and intensive care settings: a scoping review. Health Expect.

[15] Davidson JE, Aslakson RA, Long AC (2017). Guidelines for family-centered care in the neonatal, pediatric, and adult ICU. Crit Care Med.

[16] van Mol MM, Kompanje EJ, Benoit DD, Bakker J, Nijkamp MD (2015). The prevalence of compassion fatigue and burnout among healthcare professionals in intensive care units: a systematic review. PLoS One.

[17] Stricker KH, Kimberger O, Schmidlin K, Zwahlen M, Mohr U, Rothen HU (2009). Family satisfaction in the intensive care unit: what makes the difference?. Intensive Care Med.

[18] Lee HW, Park Y, Jang EJ, Lee YJ (2019). Intensive care unit length of stay is reduced by protocolized family support intervention: a systematic review and meta-analysis. Intensive Care Med.

[19] Schleyer AM, Curtis JR (2013). Family satisfaction in the ICU: why should ICU clinicians care?. Intensive Care Med.

[20] Newey CR, George P, Honomichl R (2021). Satisfaction with care and satisfaction with decision making are similar regardless of staffing model in a neurocritical care unit. Neurocrit Care.

[21] Ponsford J, Olver J, Ponsford M, Nelms R (2003). Long-term adjustment of families following traumatic brain injury where comprehensive rehabilitation has been provided. Brain Inj.

[22] Harlan EA, Miller J, Costa DK (2020). Emotional experiences and coping strategies of family members of critically ill patients. Chest.

[23] Mitchell ML, Aitken LM (2017). Flexible visiting positively impacted on patients, families and staff in an Australian intensive care unit: a before-after mixed method study. Aust Crit Care.

[24] Adams JA, Anderson RA, Docherty SL, Tulsky JA, Steinhauser KE, Bailey DE Jr (2014). Nursing strategies to support family members of ICU patients at high risk of dying. Heart Lung.

[25] McLennan M, Aggar C (2020). Family satisfaction with care in the intensive care unit: a regional Australian perspective. Aust Crit Care.

[26] Davidson JE, Daly BJ, Agan D, Brady NR, Higgins PA (2010). Facilitated sensemaking: a feasibility study for the provision of a family support program in the intensive care unit. Crit Care Nurs Q.

[27] Curtis JR, White DB (2008). Practical guidance for evidence-based ICU family conferences. Chest.

[28] Hartog CS, Jensen HI (2013). Family-centered ICU care may be good for everyone. Intensive Care Med.

[29] Cypress BS (2012). Family presence on rounds: a systematic review of literature. Dimens Crit Care Nurs.

[30] Secunda KE, Kruser JM (2022). Patient-centered and family-centered care in the intensive care unit. Clin Chest Med.

